# Dr. Duncan, Jun. on Diffuse Inflammation of the Cellular Texture, &c.

**Published:** 1825-04-01

**Authors:** 


### *Cases of Diffuse Inflammation of the Cellular Texture, with the Appearances on Dissection, and additional Observations.* By Andrew Duncan, *Jun.* M.D. &c. &c. &c.

[Ed. Transactions, vol. I., p. 470, to 670.]

We have deemed it proper to devote an entire analytical article to this paper (though an insulated communication in a volume of Transactions) for three reasons—the great length of the Essay, 200 pages—its great importance—and the great respectability of its author. The high interest of the subject cannot be questioned, when we consider that it treats of a dreadful affection, which is a constant source of danger to ourselves while engaged in pathological investigations;—and not rarely to our patients from the operation of phlebotomy.

The variety of inflammation now to be considered, is one of frequent occurrence—generally severe—and sometimes fatal. As it has not attracted commensurate attention from systematic writers, Dr. Duncan has laid his brethren under deep obligations by his attempt to collect, condense, and reduce into order, all the information on the subject which his extensive reading, and no less extensive correspondence, enable him to procure. He justly observes, that medical science has been retarded by excessive deference to the opinions of celebrated men—hence, a fashion prevails even in diseases, or rather in the names affixed to them. Those which were once considered as common, cease to be mentioned, while others, recently described for the first time, are soon found to be by no means rare. We believe with our able author, that it is highly probable, that ever since phlebotomy came into general use—and morbid anatomy to be cultivated, the same untoward accidents have happened that now frequently attract our attention. But their pathology was not understood. At one time, the mischief was attributed to the prick of a nerve, at another time, to that of a fascia—then inflammation of an absorbent vessel—or of a vein, &c. Dr. Duncan thinks, and probably with reason, "that the share which a diffuse inflammation of the cellular tissue has in these affections has been still overlooked." Our author modestly acknowledges that he may have gone to extremes, and included in "diffuse inflammation of the cellular texture," some which are of a different nature. "But such an error cannot hurt the progress of medical science, as it may lead to investigation and the establishment of truth."

Dr. Duncan divides this Essay into two sections, the first containing the facts or cases, on which are grounded the principles developed in the *second* section. This plan, which is the well-known one that Bacon long ago recommended to physicians, is the best that can be adopted, and, therefore, we shall follow our intelligent author in his own track.

*Case* 1. *From Venesection.* Hugh Snell, æt. 60, affected with diabetes, was bled in the Royal Infirmary, 8th January, 1820, and on the 12th complained of pain in the arm, which was inflamed at the puncture. *Poultice applied.* 13th. had no rest last night—arm very much inflamed, tense, and swoln. *Saline cathartic—poultice.* 14th. Consultation of surgeons. Arm much swoln, with great tension and acute pain—no red lines. The swelling and tension were uniform from the fingers to the highest part affected. *Twelve leeches—cold saturnine cataplasms.* 15th. Slept better—inflammation still advancing towards the shoulder—pulse 104—tongue white. *Twelve more leeches—cold lotions.* 16th. Slept none—swelling has reached the shoulder—head-ache and faintness—pulse 120—*sulphate of magnesia* ʒj. 17th. Leeches to the pectoral muscle, where the swelling now had reached—warm anodyne fomentations—a dark coloured bulla near the elbow, and general vesication of the surface—great œdema of the hand—complains that his throat is quite closed. Died after the visit.

" *Dissection.* The orifice made by the lancet was yet open, and readily admitted a probe, but there was no inflammation of the vein, no thickening of its coats, and no effusion into it of coagulable lymph or pus. The cellular structure of the arm was universally gangrenous, distended with sanious fluid, and the muscular fibres dark-coloured, tender, and literally rotten. The gangrene extended over the pectoral muscles of the affected side, and terminated exactly at the mesial line, forming a striking contrast with the healthy flesh of the opposite side. The lungs collapsed naturally, no tubercles, no adhesions; a small polypus, was in the right auricle of the heart, in which, and in the commencement of the aorta, there was a slight blush of inflammation. (The blush, here ascribed to inflammation, was more probably the tinging of the tissue, by the contact of blood after death.) Nothing peculiar was observed in the kidneys and renal vessels; mesenteric glands soft and cheesy; stomach greatly distended, its coats thickened; spleen blanched, and smaller than usual; liver healthy, gall bladder empty; omentum pellucid; and a general want of adeps throughout the body, as is usual in diabetic cases." 477.

*Case* 2. Ann Ralston, æt. 23, had long suffered from a succession of scrofulous abscesses, and was admitted into the

Infirmary on the 8th May, 1821, for a tense elastic swelling of the abdomen, with a sense of internal fluctuation. The urine was scanty and high-coloured—but on the 12th it suddenly became copious, and without urinous smell or taste. On evaporation it proved to be diabetic. On the 24th she was bled in the arm, the serum of the blood exhibiting a milky appearance. 25th. She passed twelve pints of urine. 26th. Was seized with pain in the bend of the arm—sickness and vomiting—pervigilium. There is a slight blush and a diffused swelling, with great tenderness round the wound—and these symptoms are all increasing—pulse 120, very full—great thirst—urine, 15 pints. *Ten leeches and a cataplasm to the arm—sulphas magnesiæ.* 27th. A bad night—constant retching—arm more swelled—pulse 130, and full—*venesection to* ℥xxiv. Blood buffy—no abatement of pain—nausea. 28th. Pain and swelling increased—some ichor discharged from the lancet-wound—frequent retching—urine scanty and turbid, with urinous smell. *One grain of opium—four ounces of wine daily—cold lotion.* 29th. Had a bad night—swelling extended to the chest, with great fulness and tenderness—a large vesication on the inner part of the arm, surrounded by a livid margin—skin underneath insensible. The symptoms went on increasing during the next two days, when death closed the scene.

"*Dissection.* On the 2d of June, at 8 *a. m.*—Externally upon the sternum and under both clavicles, particularly the right, there was much livid colour, and many small vesications. Upon the right side, immediately under the ribs, very extensive vesications had arisen, from which the water had been evacuated, and the cuticle partly removed. The same livid colour and vesications appeared upon the right shoulder, running down the back. Upon removing the integuments from the lancet-wound of the right arm upwards, a considerable quantity of serum was discharged, and some purulent matter was found in the cellular membrane which was not destitute of fat. The integuments being further removed, the muscles of the arm and chest came into view, which discovered the chief seat of the disease. The *biceps* and *pectoralis major* muscles were nearly black, and in a state approaching to gangrene; the texture, in some places, was so far destroyed, that it could be broken down by the finger; this was particularly remarkable in the *pectoralis major.* The smaller pectoral muscle was also slightly affected upon its surface.

"A probe was then introduced into the vein, which was laid open as far as the axilla. The vein was found to be perfectly healthy, and appeared not to have participated, in the slightest degree, in the surrounding disease. Its internal surface was white, and its tunics sound and healthy.

"In the thorax nothing morbid was observed.

"The abdominal viscera also were healthy, and, with the exception of the kidneys, which were thought by some to be rather larger than natural, no deviation from healthy structure was observable." 483.

It is curious that both these cases should have been diabetic—and that dissection disclosed no material lesion of any vital organ. This may be owing to the early stage of the diabetes, in which the patient was cut off, before organic changes became prominent. But the fact is, that lesions of structure are not a necessary accompaniment, much less a cause of diabetes.

*Case* 3. Michael Dogherty, aged 31, was admitted into the fever hospital on the 14th April 1821, with the usual symptoms of continued fever. Next day, he was bled to 20 ounces. The second night after bleeding was restless, and on the third day he had pain in the arm, the wound having suppurated, and the edges being everted, with a slight surrounding inflammation. On the 5th day, the pain was much increased, extending from the fingers to the shoulder—much inflammation and hardness for a space of three inches above and below the wound, from which pus could be pressed—pulse 104 and feeble—much febrile heat—countenance anxious and oppressed. 7th day, inflammation extended upwards and downwards—constitutional symptoms continue. 11th day, the whole arm inflamed and œdematous, with great pain on the least motion—a rigor in the preceding night, followed by delirium—great uneasiness across the chest—stools dark. 13th day, constant sighing—delirium—pain of chest—feet and legs œdematous—difficult articulation. 24th day, a large sphacelation over the sacrum—other symptoms the same. 27th day, the wrist of the other arm has swelled, and there is a distinct fluctuation in it. 30th day, terminated his earthly career.

"*Dissection.*—The body was much emaciated. On removing the integuments from the right arm, the subjacent cellular tissue was found considerably altered in structure, being converted into a firm ligamentous-like substance, and with difficulty separated from the surrounding parts. The *mediana longa*, from the back of the thumb to its termination in the median cephalic and basilic, presented the appearance of a nerve, being somewhat of a fibrous structure, but resembled an artery when cut transversely, the cut extremities presenting circular orifices, and not collapsing, as in healthy veins. When slit open, its coats were greatly thickened, and its surface lined with a thick layer of coagulable lymph. About the middle of the fore-arm, a small abscess formed in the vein, but was prevented from extending by the coagulable lymph. Above two inches above the bend of the arm, the cephalic and basilic veins were filled with pus, and their coats were uncommonly thin and easily ruptured. This appearance extended to the axillary vein, and terminated abruptly before the vein crossed the first rib. The vein remained quite pervious, though its cavity was much diminished, neither did it contain a particle of blood, nor could its valves be observed. Between the first and second ribs, near to their sternal extremities, an opening was discovered leading into a sac, formed by an adventitious membrane of coagulable lymph, filled with purulent matter, and pushing inwards the *pleura costalis*. The sac contained four or five ounces of pure pus. At the left wrist there was a similar collection of matter, containing about six ounces of pus. The lungs were perfectly healthy in their structure, but several old adhesions existed between the pleuras in both sides. The heart was natural and healthy; but on the left side there was a deposition of coagulable lymph on its external surface, and the inner coat of the aorta to its arch had a deep red appearance. The other cavities were examined, but nothing of any importance was found." 486.

Cases 4, 5, 6, of the Essay were severe but not fatal, from venesection; we shall therefore pass them over, and come to case vii.

*Case.* 4. Mary Macgregor, ætat. 58, had had the right mamma successfully removed for scirrhus ten years previously, and was re-admitted on the 1st September, 1821, for a scirrhous tumour in the axilla of the same side. This was removed on the 8th, and a vein was tied in the operation. 9th, Had a rigor in the night, followed by fever this morning—complains of pain of breast and of arm—vomits all liquids. 10th, Gastric irritability continues, with pain at the ensiform cartilage. Constipation. Leeches to the epigastrium. 11th, Nausea sine vomitu—tongue foul and dry—pulse 108—no stool since the 8th—thin unhealthy discharge from the wound. These symptoms continued till the 16th, when the patient died.

"*Dissection.* The parietes of the cavity left by the tumour were found in a sloughing state. The neighbouring muscles, particularly the *pectorales*, *teres major*, and *latissimus dorsi*, were quite black, and yielded to the slightest violence. In their interstices there was much effusion of a yellow substance, resembling coagulable lymph. A ligature was found round the axillary vein, about half an inch below the entrance of the cephalic, and another upon the subclavian, a little above the entrance of the cephalic. A clot, about 1½ inch long, was formed in the axillary vein, below the ligature, and a firm yellow fibrous clot, not quite so long, in the subclavian, above the ligature. The internal coat of the vein below the ligature seemed inflamed. The coats of the vein, from the upper ligature to the entrance of the external jugular, were much thickened, so as to resemble the coats of an artery. The portion of vein between the ligatures was much shrunk on the side next the axilla, and a small puncture was distinctly visible between them. The coats of the ascending cava, right auricle and ventricle, were very dark coloured." 494.

This brings us to cellular inflammation from punctures and cuts received in dissection; but as we have published so many cases of this kind in our Journal, we shall select but a very few from the Essay before us, and those chiefly of fatal issue, where dissection rendered the cases more perfect.

*Cases* 1. *and* 2. Two medical gentlemen (Messrs. Blyth and Young) examined the body of a young woman on the 28th December—the body presenting no uncommon appearance externally, emitting little or no putrid smell, and being very emaciated. There was much sero-sanguineous fluid in the chest, in removing which, with a tea-cup, Mr. Blyth's hand came frequently in contact with it. Mr. Young put his hand only once into the thorax, to squeeze the lungs. In a few hours after the dissection, Mr. B. felt a slight smarting on the forefinger of the right hand, and, on examination, perceived a very slight abrasion of the cuticle, not larger than a pin's head. He applied a bit of sticking-plaister, and thought no more of it that day. Next morning, on going out he felt very chilly, though the air was very warm for the season. Throughout the day he felt languid, and depressed, and towards evening became quite ill. Dr. W. Campbell was called in, and immediately bled him from two veins, but very little blood could be obtained.* In the same evening a lancinating and excruciating pain took place in the glands of the right axilla. On the same evening Mr. Young became affected with the symptoms which usually characterise typhus fever. He also had an abrasion of the cuticle in one of his fingers, but there was no appearance of surrounding inflammation, tenderness to pressure, or pain. In his axilla, as in that of Mr. Blyth, was the first appearance of local affection. From the glands of the axilla, in both cases, there was a diffused and painful swelling, with considerable redness up along the neck, and for several inches down along the side. The swollen parts did not pit on pressure, but gave an obscure sense of fluctuation, which has been well characterised by Mr. Lizars by the term *boggy*. A lancet was

* We have often, in the course of this Journal, and in our practice, objected to the principle of blood-letting in the forming stage of fever, when there is chilliness, depression, and where the re-action has not fairly set in. It is a good principle to bleed *early* in fever, if you are to bleed at all. But surely this applies to the re-action of fever, and not to the languor, chilliness, and depression which generally precede such re-action plunged into the swelling on Mr. B's side, but nothing except a little blood issued. A number of leeches were now applied to the parts affected—and a blister to the thorax, which did not rise well. Mr. B. became very restless, without delirium, which state continued several days and nights. Anodyne antimonial draughts failed to procure sleep, and the patient suffered much from thirst, which no drink could quench. This state continued eight days, the diffuse swelling extending down the side, with increased redness of the skin. The pain and inflammation then gradually diminished—the cuticle peeled off, and they were congratulating the patient on his fortunate escape, when one morning a quantity of pus was seen to issue from the puncture made in his side at the beginning of his illness. An abscess was now discovered, extending as far as the ilium, and nearly to the pubes. Several punctures were necessary for the exit of matter. Much matter, mixed with shreds, continued to discharge for many days. He appeared to be recovering rapidly, when an apthous sore mouth took place. "The whole of the fauces, as far as they could be seen, was encrusted with a thick layer of coagulated albumen, which was no sooner detached than it was renewed." Deglutition was very difficult and painful, and the secretion of viscid saliva was excessive. A blister to the throat was the only thing that gave any relief. An alarming and distressing hiccup now occurred, which was at last removed by sipping small quantities of milk and water. At length convalescence was established, and health restored.

Mr. Young's case, though strikingly similar in its commencement and early progress, was not so fortunate in its termination. The symptoms and treatment were the same as in the case just related, and recovery was confidently expected when Mr. Y. was suddenly seized with a high degree of pleuritis that did not yield to venesection. In the evening of the same day he became outrageously delirious, and died next morning, 4th January, 1822.

"*Dissection.* An incision was first made from the clavicle to the crest of the os ilium, and another crossing it to the axilla, when the subcutaneous cellular substance appeared in some places distended with serum, especially about the loins, while in others it was turgid with purulent matter, which was most conspicuous over the *pectoralis major.* On prosecuting the dissection, the whole cellular substance of the side, from the axilla down to the os ilium, and from the spine to the sternum, was more or less purulent; the cellular sheaths of the *latissimus dorsi, serratus major anticus, pectoralis major* and *minor* muscles, were all purulent; and even the prolongations of the cellular membrane, which divide the muscles into their different fasciculi, were equally purulent. Between the two pectoral muscles, and beneath the *minor*, the infiltration of the cellular substance with purulent matter was very conspicuous. The carneous fibres of these muscles had in general lost their cohesion, and were of a dirty-yellow colour. The intercostal muscles were pretty sound, but the external surface of the pleura beneath them was highly inflamed, and the vessels bold and distinct, and conspicuously ramified.

"In the axilla there were numerous small collections of purulent matter, as if it had been secreted into the cellular tissue. The lymphatic glands were enlarged and inflamed, but had not suppurated. The nerves possessed their natural colour, so did the axillary artery, and even its branches, with the exception of those immediately supplying the axilla. The vein in some parts had a dirty-red appearance, and the small branches coming from the axilla were immersed in purulent matter, and had lost their tenacity.

"The dissection was next carried down the arm: the brachial vein, as well as the median-basilic, was slightly red in several places, and a twig of the internal cutaneous nerve presented an inflamed appearance; which, however, ceased at the bend of the arm. The cellular substance of the arm was every where healthy, and there was not the slightest vestige of disease in the fore-arm, nor could any connexion be traced between the abrasion on the finger and the morbid parts.

"The thorax was now examined: the pleura of the right side, which has been already mentioned as inflamed on its outer surface, was much more so internally. Here its colour was deeper, and the inflammation was in patches, extending over the whole surface of the cavity to the mediastinum and pericardium. The surface of the right lung had a white appearance, from coagulable lymph effused upon its *pleura pulmonalis*, which was considerably thickened, but the substance of the lung itself was healthy. In the cavity of this side of the chest, more than three pints of bloody serum, with flakes of coagulable lymph floating in it, were found. The pleura and lung of the left side were perfectly healthy, but some bloody serum was found in the cavity, though not nearly so much as on the right side. The *vena cava descendens*, and the right auricle of the heart, appeared inflamed, but the other cavities were healthy, and all of them filled with blood, part of which had an appearance of fibrine. The walls of the ventricles, when cut across, were darker than natural." 502.

Passing over some cases which were not fatal, we come to a very short notice of Dr. Dewar's melancholy end. This gentleman pricked a finger of his *left* hand slightly, while examining the body of an enteritic patient, on the 13th february 1823, and immediately applied caustic to the wound. On the 14th he remained in bed, complaining of languor, general debility, and rigors. On the 16th, the *left* axilla became exceedingly painful, without much swelling of the part, or any hardness in the course of the absorbents on the arm. Leeches were applied to the axilla, with great relief to the pain. On the 18th, one of his medical friends was sent for, on account of an accession of pain in the *right* fore-arm, with some swelling and redness. Leeches were immediately applied, but without affording any relief, for the inflammation and swelling extended rapidly. He was bled from a vein, and the blood was sizy. Early in the morning of the 19th, he became insensible, and died in the evening of the same day. "The symptoms throughout were those commonly called typhoid." The body was not examined.

A curious case follows, which our author says was *not* fatal, though the patient died on the 11th day of the disease.

Mr. Cumming, a medical practitioner, was a spectator at the dissection of a young woman who died of puerperal fever. He did not touch the body, and only introduced a fresh thread into a needle employed in sewing up the abdomen. He was not aware of any puncture or abrasion. About eight hours afterwards he felt an uneasy sensation in the middle finger of the *left* hand, at the inner side of the flexure of the first joint, where, on examination, was found an angry pimple. He passed a restless night, and towards morning had a severe rigor succeeded by pyrexia. Next day (1st Oct.) he was visited by Dr. Fairbairn, who observed a sero-purulent fluid oozing from the pimple. A poultice was applied. The following day, (2d Oct.) the parts about the axilla and under the pectoral muscle were greatly swelled, and painful on being handled, conveying an elastic feeling to the finger, accompanied by fever, violent headache, and great constitutional disturbance. He was bled four times from the arm—had 24 leeches applied twice to the shoulder—diaphoretics and cathartics internally—anodyne astringent lotions externally. Under this treatment, the symptoms greatly subsided in four or five days—and the swellings were so much reduced, that the patient could move his arm freely. On Saturday evening, the 7th of October, a deep-seated swelling, the size of a pigeon's egg, was discovered in the *other* arm, between the wrist and the elbow, which was circumscribed, with some redness and pain on pressure. 8*th*. The swelling had increased considerably, occupying nearly the whole fore-arm, with an erysipelatous appearance. The pulse, at the wrist, could be but obscurely felt. 9*th*. The tumefaction has increased to an alarming extent, reaching from the fingers to the *acromion scapulæ*. The parts are evidently gangrenous. 10*th*. Death took place this day. The body was not examined.

The next case is remarkable, when taken in connexion with the preceding. A towel, which had been used instead of a sponge during the dissection, which proved fatal to Mr. Cumming, was washed in the evening by Mrs. Edie, who got one of her fingers scratched by a pin in the said towel. She felt the part painful next day—and had shivering, head-ache, and pyrexia. Inflammation and swelling soon extended from the finger up the arm, attended with excruciating pain. *Venesection—a dose of salts.* On the 4th day, she was seen by Dr. Fairbairn, when the inflammation and swelling were as high as the elbow, and had an erysipelatous appearance. Several glands were also enlarged and painful. The constitutional symptoms were severe. *Venesection twice—diaphoretics and cathartics.* Under this treatment the inflammation was partially checked, and the fever somewhat subdued. Matter at length formed in the palm of the hand, and was discharged by incision. She recovered.

We must now pass on to the second Section of the Essay, containing "a general account of the diffuse inflammation of the cellular substance," commencing with—

I. *The Etiology. Venesection* is the first cause which is commented on by Dr. Duncan. Instances, satis superque, are cited from Ambrose Paré, Hildanus, Dionis, O'Halloran, Abernethy, Charles Bell, and others, to shew how frequently phlebotomy is followed by this intractable and dangerous species of inflammation. *Ligatures to veins* is the next cause enumerated, and the subject illustrated by reference to the cases published by some modern authors, especially Sir Astley Cooper, and Mr. Travers. *Dissection-wounds,* however, have proved to be the most fatal if not the most frequent source of the inflammation under review. The cases already stated—and those published in various journals and transactions, are adduced as satisfactory proofs on this point. *Inoculation by morbid secretions of living animals,* is brought forward by Dr. Duncan, as another source of cellular inflammation, and cases are adduced from Chaussier, Sir E. Home, Dr. Patrick Russel, and others, in illustration. *Acrid matters* directly applied to the cellular substance, appear, from the experiments of Orfila, to be capable of exciting this peculiar species of inflammation. In fact, pricks and wounds, without any morbid poison—sprains—external violence, are all capable, in some cases, of giving rise to the disease—and, lastly, it has arisen spontaneously, or, at least, without any known cause. *Erysipelas phlegmonodes* is very properly considered by Dr. Duncan as one of diffuse inflammation of cellular tissue, of which we have seen numerous instances, and many of them fatal.

*Phlegmatia dolens,* our author thinks, must be explained on the principle of diffuse inflammation of the cellular structure, as ably maintained by Dr. Hull of Manchester.

"Indeed, those cases which have been examined after death, shew the same destruction of cellular texture, the same extensive suppuration that we observe in *erysipelas phlegmonodes*; so that these diseases differ chiefly in their exciting cause, and in the total absence of cutaneous inflammation in most cases of *phlegmatia dolens*.

"This view of the pathology of this disease has been strikingly confirmed by the dissection of a case by Professor Casper, in which the cellular membrane was extensively inflamed, *and the blood-vessels, on careful examination, ascertained to be without disease*." 583.

Did this case of Casper's elude the erudite researches of Dr. Davis? Certes he ought to have noticed it, although it goes directly against his theory. Casper distinctly says:—"Percaute reteximus vasa magna sanguifera, omnino verò sana visa sunt et in externa et in interna superficie"* The cellular membrane was diseased throughout the whole limb, and even in the pelvis,

"It is more probable, that the disease partakes of the nature of an epidemic. At least, severe and fatal cases have been more common of late, than at any preceding period. Nor can I ascribe this to the disease having been described under another name, or having escaped observation, for the symptoms are too severe, and the death, when it happens, too sudden to be overlooked; and yet we find few such cases recorded under any denomination.

"The causes why this disease should be more frequent at one period than another, are quite unknown to us. It does not seem to be connected with temperature, or any of the ascertainable atmospheric states; for it has occurred at all seasons of the year. Nor can it be ascribed to any deficiency of wholesome food. It is, however, worthy of remark, that severe and fatal cases occurred when other congenerous, though slighter diseases, were very common." 586.

Thus, in Edinburgh, for the last four or five years, a great disposition to erysipelas has been observed, not only in hospital, but in private practice. Our observations, as Dr. D. observes, are too few to enable us to draw any conclusion as to the influence of situation, and those causes which produce endemic diseases. It is generally supposed to occur more frequently in large cities than in the country—and more frequently in crowded hospitals, than in other situations.

Upon the subject of the *predisposing causes*, Dr. D. has little to adduce. Some cannot handle the viscera of a recent body with impunity, although there be no breach of structure in the skin; while others have often pricked and cut themselves without any bad consequence. Leech-bites always fester on some

* Commentarius de Phlegmatia dolente. Auct. J. L. Casper. 8vo. Halle, 1819.

skins, while other surfaces can scarcely be affected by a sinapism. Who can determine the nature of these idiosyncrasies, or foretel them? None.

*Symptoms* These differ in different individuals. In a few instances, the disease commences with constitutional symptoms, of the typhoid kind, and the local affection does not shew itself for two or three days. But, in the majority of cases, the local precedes the constitutional affection—being sometimes confined to the limb, but at others, proceeding rapidly to the trunk, and terminating fatally.

"In a few of the cases induced by venesection, the lancet-wound seems to heal as readily as usual. In some it remains permanently united, but in others it opens again, or at least, the wound of the skin gives vent to the discharge of some purulent matter. More commonly, union does not take place. The lips of the incision remain swollen, a little red, and everted. Some ichorous or purulent discharge appears, and the local disease extends continuously from the wound." 593.

In the most alarming variety, the affection follows the usual progress of inoculated diseases First, a pustule or vesicle takes place at the part where the poison is applied—then there is high constitutional disturbance, corresponding to the eruptive fever of the exanthemata, followed by severe diffuse inflammation in some parts of the cellular tissue, rapidly extending in all directions, but not continuous with the primary local pustule, which often gives little uneasiness, and generally heals very quickly. In what way the affection is propagated from the local injury on the fingers to the part which is the principal seat of the secondary disease, is very doubtful. There is no evidence that it is exclusively by phlogosis of the veins or absorbent vessels, as some have maintained. When the disease proceeds from inoculation, the constitutional symptoms generally occur in the course of one or two days from the accident.

*Secondary Inflammation.* This is generally in the axilla, when the cause is applied to the hand or arm. Thence it extends towards the sternum, up along the neck, or downwards along the side. In most cases, it is confined to one side—but sometimes it passes the mesial line, and invades the opposite side of the body. In other cases, again, it is translated from one side to the other, not by any regular spreading of the inflammation, but by metastasis, as we see in gout, rheumatism, and erysipelas.

"When the swelling reaches the axilla, whether it advance to it progressively from a lancet-wound in venesection, or appears at once after dissection, it is always of a very peculiar and characteristic nature. It is diffuse and extensive, without the slightest tendency to point, being only flatly elevated above the neighbouring sound parts, and its limits are rarely defined by a margin, which, however, is sometimes raised. It is smooth and equal, having no central hardness, no *focus* where it is more active. If the glands can be felt enlarged, it is only in a slight degree; and, in general, no cords which can be supposed to proceed from enlarged or thickened lymphatics, veins, or other vessels, can be traced under the surface. But it is the general feeling and sensation which the swollen part gives to the touch which characterises it, and which depends upon the effusion of fluid generally into the cells of the cellular membrane. It is tense, but soft in a greater or less degree, and when pressed by the finger, does not pit, but gives a sensation between the resisting hardness of phlegmon, the yielding softness of œdema, and the elasticity of emphysema. The epithet of *boggy*, applied to it by Mr. Lizars, appears to me exceedingly expressive of its nature. Like a quagmire, it conveys the idea of a firm surface, with a spongy, unsound bottom, loaded with fluid." 598.

This obscure sense of fluctuation has led many, and among others, Dr. D. himself, to make deep punctures, with the view of giving vent to a supposed collection of fluid, when there was nothing to be discharged.

The *pain*, in almost every instance, is represented as exquisite, and often precedes any redness of the part. In some cases, the secondary inflammation runs its course, terminating in extensive suppuration, without any redness of the skin being perceptible—"and in all true cases of this disease, the cutaneous inflammation is secondary, and the result of the progress of the disease from the cellular tissue to the skin." Thus, in the hospital nurses, there was no affection of the skin, although the existence of most extensive cellular suppuration and gangrene was positively ascertained in one of them by dissection.* Vesicles or bullæ very seldom appeared till the disease was far advanced; nor has Dr. Duncan observed the vesicular-like tubercles which were seen in the case of Mr. Hutchinson, and the late Professor Dease; but all his observations confirm, in the strongest manner, Dr. Colles's observations on the slight connexion between it and the affection of the skin, and the total difference of the disease from erysipelas.

---

* In an epidemic of this kind, which once raged among the crew of the Royal Oak, in Basque roads, and where several cases proved fatal, we have seen the whole cellular substance of the thigh, for instance, so completely destroyed (without any thing visible on the surface) that when the integuments were slit up, the whole would fall off, leaving the muscles underneath as clean as if they had been dissected by an expert anatomist. The men that died, all exhibited symptoms of typhoid fever. *Rev.*

There is something remarkable, also, in respect to the temperature of the parts. It is often below par. This is particularly remarked by Mr. O'Halloran, where the swelling is represented as œdematous, cold, and insensible. Sir E. Home was also struck with the coldness of a man's arm bit by a rattle-snake. Such a degree has not been observed by Dr. D. but it may be explicable by the sudden effusion which takes place into the cellular tissue, diminishing the connexion of the skin with the sources of animal heat.

"*Fever.* In most instances, the affection was accompanied with excessive constitutional irritation, and a very high degree of fever, which well deserved the appellation of typhoid, from the extreme muscular debility and depression of mind which accompanied it. Indeed, on some occasions, it was scarcely possible to determine, whether it was a case of this disease, or of typhous fever with peculiar local symptoms.

"The symptoms and progress of the fever presented considerable varieties. It sometimes commenced insidiously, sometimes tumultuously, but, in most of the severe cases, soon reached its height. The chief peculiarities I observed were the supine posture with depressed shoulders, in which the patient almost always lay, without turning to either side, the absence of coma, and the rare occurrence of continued delirium." 603.

Some of Dr. D's patients emitted a peculiar cadaverous smell during life—and, in one case, a fetid and coloured sweat proved critical.

"My notice was strongly attracted by the morbid state of the respiration in several of these cases; and, in fact, there are two very sufficient reasons for it; one of which almost always occurs, and both not unfrequently. When we consider that the inflammation, with extreme tenderness most commonly affects the *pectoralis major* and *minor*, *serratus anticus*, and other muscles connected with the ribs, we may be satisfied that their motion must give uneasiness and pain; but when the intercostals themselves are involved in the disease, their action becomes more distressing. The pain in these cases resembles that in an exquisite degree of pleurodyne." 603.

In some cases, however, the pain is not solely muscular; the inflammation extends to the pleura, and produces actual pleuritis.

*Terminations.* In the very slight cases only, when arising from an injury, does diffuse inflammation of cellular tissue, terminate in resolution. If our author, however, is right in considering acute anasarca and phlegmatia dolens as examples of this disease, we have resolution a very frequent termination in such cases. The next most fortunate issue is abscess; in which case, the effusion of coagulable lymph seems to arrest the extension of the inflammation, by producing accretion and obliteration of the cells, and concentrating, as it were, the inflammatory action within a limited space, converting the diffuse and spreading, into the fixed and circumscribed phlogosis. This termination is not unfrequent, after venesection, when abscesses form near the edge of the biceps muscle, or in the forearm. Diffuse and extensive suppuration is a more common termination in severe cases, and is observed both in those which recover, and in those who die.

"When the recovery after suppuration and sloughing is complete, the cellular membrane seems to be regenerated; and this is the more probable, as we see it every day formed in these preternatural adhesions which take place between serous surfaces, and in what are called new or false membranes.

"In some cases, however, Nature seems to be inadequate to the reproduction, or rather the state of the neighbouring parts necessary for the reproduction has been permanently affected; and we find adhesion of the skin to the subjacent muscles, or deeper seated adhesions remaining, giving rise to permanent contraction of the limbs, or rigidity of the muscles. In many instances, these gradually disappear, in a great degree, but in others they are more or less permanent. This seems especially to be the case, when the diffuse inflammation of the cellular tissue is complicated with that of the skin." 606.

When this inflammation extends to the cellular tissue forming the attached surface of serous membranes, as the pleura, &c. the whole serous membrane soon becomes affected, and gives rise to corresponding phenomena. Transition to the mucous membranes has not been so distinctly observed in this disease; yet, in one case, that of Mr. Blyth, an aphthous inflammation was very troublesome during convalescence. Even the osseous system does not always escape the ravages of this formidable disease. Our author found, in one case, the ends of several of the ribs carious, and denuded of periosteum. In other instances, there was reason to believe, that the periosteum was included in the disease. The period of fatal termination varies, from two or three days to as many, or more, weeks.

*Post-mortem Appearances.* Dr. Duncan gives a methodical resumée of the appearances on dissection; but, as we have quoted very freely the dissections themselves, in their proper places, we shall be excused resuming the subject, with the exception of a single quotation:

"As this disease is progressive, affecting one part first, and then others in succession, we find it after death existing in different parts, in all its stages. Accordingly, in the part most recently affected, which was often the space between the twelfth rib and the os ilium, we find the cellular substance merely œdematous, with increased vascularity; the serum is still fluid, and limpid, or tinged with red, and readily flows from the divided tissue. In a more advanced stage, the effused matter is less fluid, often higher coloured, and has not yet acquired the opacity and whiteness of purulent matter. We next find the cellular membrane gorged with a white semi-fluid matter, which does not flow from the incision, but greatly augments its thickness, and separates the particles of fat to an unusual distance from each other. In the subsequent stage, it continues opaque, whitish, or reddish, or greenish, but becomes more fluid, so that now purulent matter flows from the incision. But the pus is still contained in the cells of the tissue; and it is only in the last stage of the disease, and after the tissue is entirely broken down, that we meet with collections of purulent fluid, with sloughy membrane; even then, however, the pus is not contained in a cyst or circumscribed cavity, but is gradually lost in cellular substance in the preceding stage of the change, without any line of demarcation.

"When fluid pus is formed, I have already spoken of it as if it were broken down cellular substance; but it is perhaps rather a secretion, which is in such abundance as to rupture the cells, and break down the cellular membrane, so that the portions thus disjoined from their necessary attachments become dead, or, in common language, sphacelous. In this way we may account for the masses, like skeins of thread, drawn out of Mr. Blyth's side; and the sloughs of cellular membrane, described by Sir E. Home as resembling wet tow, and by Mr. James as looking like large wads of wet shamoy leather." 610.

*Diagnosis.* When we consider how extensively the cellular tissue enters into the composition of all our organs, it is difficult even to imagine a case perfectly free from complication. Still we do not think that any observant practitioner will confound phlegmonous with diffuse inflammation—and still less this last disease with erysipelas or inflammation of the skin. From phlebitis, it will be generally distinguished by the cord-like feel of the vein, and the tenderness on pressure being confined to the track of the vessel. In some cases of phlebitis, however, there is considerable difficulty in distinguishing the two diseases, nor will any verbal discriminations be likely to assist us.

*Prophylaxis.* As some people are much more than others obnoxious to the bad consequences of dissection-wounds, they ought to wear gloves in their *post-mortem* investigations, where no great dexterity with the knife is at all necessary. It is a proper precaution, Dr. Duncan thinks, to anoint the hands well with camphorated oil or simple lard, before handling the viscera. The precaution of examining the hands to see if there be any abraded surface, before dissection, is too often neglected—though it is a very necessary one.

*Local Treatment.* The indications are three—to remove the poison before it has time to operate on the system—to render it effête—or enable the system to resist its impression. For attaining the first object, Dr. D. observes that, "nothing is more effectual than instantly sucking the wound with the utmost force, by which means the poison is often removed, and no fear need be entertained of any bad effects upon the mouth which receives it." This we grant, provided there be no abraded surface in the mouth itself—a thing not very unusual. Celsus recommends a cupping-glass to be applied—but this can seldom be done, as, for instance, when the finger is wounded. The next, in point of simplicity, is careful ablution of the part by hot or cold water—or both in succession. The destruction of the poison, or rather of the parts to which it is applied, is often attempted by chemical agents, as nitrate of silver, the mineral acids, or caustic potash. With some of these the dissector should be provided. "But of all the means of destroying the properties of the poison, none is equal to the actual cautery or excision, when practicable."

For enabling the system to repel the poison, powerful stimuli to the part have been recommended. Dr. Colles directs that the anatomist should plunge his finger into a cup-full of *ol. terebinth.* the moment it is wounded, in the hope that the irritation may counteract the power of infection, or alter the mode of inflammation in the wound. This practice is often employed by country-people to punctures from pins or needles to prevent festering. Dr. Duncan seems to entertain a favourable opinion of ligatures above the wound, with the view of preventing the absorption, or of setting a limit to the extension of the disease, when once excited. "The judicious application of a ligature by Mr. Burton, in his own case, seems to have set a limit to the extension of the disease towards the trunk, and was probably the means of saving his life."

When the poison once acts upon the system, the effects are of two kinds—local and constitutional, for each of which appropriate remedies are to be sought. With the view of reducing the local inflammation, Dr. Duncan has found the continual application of a considerable degree of cold eminently beneficial, so long as no phlegmon had formed—for, after that period, cold seemed prejudicial, and warm fomentations useful.

The patient's own feelings will always be an index on these occasions. Local bleeding by leeches, or scarifications from the part are never to be neglected, in the first instance, with the object of setting up a counter-irritation on the surface, and diverting the inflammatory action from the internal and more important tissues. It is upon this principle also, that the action of blisters is frequently of service in the early stages—although, at a later period, their salutary action may be explained by the large discharge of serum which they produce. It is upon the same principle that the application of the actual cautery may prove serviceable. This was put in practice, and with good effect, by Morand. In none of the cases which came under our author's notice did the leech-bites shew the least tendency to fester or become gangrenous.

"As soon as any fluid seems to be effused into the cellular tissue, which in the severe cases is very soon, I am convinced that the best practice is to give it immediate vent by free incisions. When, deceived by the sensation of deep-seated fluctuation, we content ourselves with plunging in a lancet into the part, we are disappointed by no discharge taking place; still the puncture sometimes proves of essential service, by facilitating the discharge, as soon as the effused matter becomes sufficiently fluid to flow out. Thus, the deep puncture made in Mr. Blyth's side appears to have been the eventual means of saving his life.

"But a much bolder practice is preferable, and one which was long ago recommended by Mr. O'Halloran, on the sure ground of experience. He gives an account of the treatment of three cases, in all of which the disease followed venesection. In the first case the arm was scarified lightly in several places, but the patient died on the 9th day. The second case was seen by Mr. O'Halloran on the 5th day after, and again on the 8th. He now resolved to make "many profound incisions the length of the arm, in hopes that the activity of the stupes and poultices would sooner pervade these parts." Wherever he made his incisions he found the adipose texture swelled and spongy, so much, that at the depth of an inch he did not cut through it, shewing that little danger accompanies profound incisions in such a state. Water, clear as crystal, started from the wounds, and was increased by pressing on the contiguous parts. His first incisions were made near the bend of the arm, and round it, in nearly parallel lines, each above an inch long, and about half an inch distant. In about two hours he made a similar range of profound incisions, about two inches higher up the arm, and next day a third and fourth set; the swelling of the arm returning between each. At night the arm was visibly lessened, and on renewing one of the incisions over the biceps muscle, about a table-spoonful of pus, of a pale whiteness, spouted out of the orifice, and, for the first time, the patient sensibly felt the heat of the stupes, and the activity of the dressings, which, he observed, produced a tingling heat all over the arm. Next day more profound incisions were made, and on opening the sores at night, those of the arm which before seemed so deep and extended, now appeared small and superficial, on account of the subsiding of the swelling of the skin and *corpus adiposum.* After this he speedily recovered his health, and the use of his arm and fore-arm. The dressings applied were of the most stimulating nature, and he got a liberal allowance of wine and generous diet." 641.

The treatment by deep scarifications, employed by Mr. A. Copland Hutchison and others in erysipelas phlegmonodes (which is neither more nor less than the disease now under consideration) is strongly in favour of that just recommended by our author, and put in practice long ago by Mr. O'Halloran.

*Constitutional Treatment.* The principles of the general treatment in this dangerous disease are very far from being settled, as the following brief sketch of the various plans tried or suggested by different writers, will testify.

Celsus prescribes early stimulation—as wine and spices. Dionis combines stimulation and depletion. He says, we must bleed plentifully from the opposite arm, and administer cordials internally. Mr. O'Halloran bled his first patient, gave him an active warm purge, and afterwards exhibited the bark in substance, with strong broths—but the patient died. His second patient was also bled, had a warm purge—then strong port-whey, claret, and strong seasoned broth. He recovered. The third patient had an irritable stomach. She was purged smartly by two grains of calomel, and next day took the decoction of bark with sulphuric acid, which the stomach constantly rejected. She survived, but was paralytic. In conclusion, Mr. O'Halloran states that, although he made use of blood-letting in his first two cases, yet, upon mature consideration, he condemns it, " as rather weakening nature than assisting us," except upon very urgent occasions. He recommends warm purges, seasoned broths, wine, and jellies, &c. Mr. Abernethy scarcely notices the general treatment—nor is Mr. Travers more particular. His patients, however, seem to have been bled and purged. Dr. Colles found large doses of opium ineffectual in relieving pain, nor did the usual remedies make any impression on the fever with which Mr. Hutchison was affected. In the more advanced stage his strength was supported only by large quantities of wine. Professor Dease was bled, purged, had laudanum, ammonia, wine-negus, and cordials—but in vain. It is almost needless to say, that all the specifics which have been extolled against the bites of poisonous animals are of the stimulating or cordial kind. We now come to Dr. Duncan's own sentiments respecting the constitutional treatment.

"The cases which have fallen under my own observation, have been treated almost entirely antiphlogistically, and in the greater number the lancet has been repeatedly employed. Both from the relief, which has often been immediately given, as well as from the indisputably inflammatory nature of the disease, there remains no doubt in my mind as to its propriety in most cases; and although it has failed in many, this must be ascribed to the intensity of the disease, which no depletion, consistent with the continuance of life, is able to overcome. Thus Mr. Cumming was bled no less than four times, besides the repeated application of leeches, but in vain; and the case recorded by Dr. Le Herissé shews, that very copious abstraction of blood is unable to prevent or mitigate the disease in every instance, for the patient was bled on account of epilepsy, on the 1st November, twice from the arm; on the 8th from the foot; on the 10th and 13th from the jugular; and on the 16th from the arm, and this puncture was the cause of the disease: to cure the disease, on the 18th the temporal artery was opened, and blood freely abstracted, but he died on the 23rd.

"The blood drawn has in general been remarkably sizy, some describing it as covered with a yellow crust, and others comparing it to calves-foot jelly. This appearance is owing to a very complete separation of the fibrine and red globules, while at the same time the fibrine does not contract and express the serum, as it does in what we call cupped blood.

"Thus we see, that, in the treatment of this disease, very opposite means have been employed, and both appear to have been occasionally successful, and both have failed. But their salutary effects depend upon different principles. In general, by the antiphlogistic treatment, we are enabled so to lessen the existing inflammatory state, that the system is capable of recruiting and performing the office of restoration to health; but, on the other hand, by the free exhibition of diffusible stimuli, we seem, in some instances, to impart to the system such a degree of preternatural energy, as to resist and extinguish the diseased action, after which health is again restored by gradually abstracting the cause of the artificial and salutary excitement. In some cases, however, we are perplexed by opposite indications, when an extreme degree of debility requiring cordials, exists along with intense inflammation, which can be conquered only by further depletion. In these cases, we must have recourse to the alternate, or even simultaneous, employment of the most opposite remedies." 648.

This is probably as judicious advice as can be given. From what we have seen of this dangerous disease, we confess that our opinion of depletion has not been greatly raised, but on the contrary, we much doubt its propriety, excepting in its local application. The tumult occasioned in the system by the introduction of a poison is not like that of an inflammatory fever, dependent on strong inflammation of an internal organ, nor is it likely to be subdued by antiphlogistic remedies alone. We quite agree with Dr. Duncan, however, that depletion and stimulation are by no means to be always considered as antagonising remedies. In many cases, it may be advantageous to diminish the quantity of circulating fluid, and excite the living solid to greater action, at the same time. We shall conclude this paper with the following passage which also concludes the Essay.

"Hitherto I have considered the debility we have to combat, as the effect of preceding excitement exhausting the powers of life; but, in the very worst cases, no symptom of excitement precedes the appearance of extreme debility, which may thus be considered as primary. It may be said, that in such cases, the cause, whatever it may be, has operated on the constitution as a direct sedative, reducing its powers, and rendering them incapable of reaction. But this explanation is equally inconsistent with facts; for if the patient survive a certain length of time, this state of debility and depression is succeeded by a very high, but peculiar, state of excitement, producing in its turn true exhaustion, and often irrecoverable debility. In fact, the state of excitement in every inflammatory disease is preceded by a state of depression. This is universally acknowledged, and, *post horrorem*, forms a part of every definition of febrile diseases, but still the importance of the cold fit has not been sufficiently appreciated, except in the case of ague. It must, however, be admitted, that in the pure *phlegmasiæ*, the cold stage is short and transient, and the hot stage long and durable. Hence our treatment is chiefly, or almost solely, adapted to the state of excitement. But it appears to me, that the malignant form of all inflammations, such as occurs in some instances of diffuse cellular inflammation, commences with an intense and long continued cold stage, in which the patient may be carried off before reaction is established.

"According to this view of the disease, we should treat those malignant cases, which begin with the symptoms of extreme debility, on the same principles that malignant intermittents are treated during their dangerous cold stage; that is, we should administer diffusible stimuli freely until the commencement of reaction, when the antiphlogistic treatment should be vigorously enforced." 650.

We need make no apology to our readers for the length of this analysis. The importance of the subject, the obscurity of its pathology, the difficulty of treatment, and the ability of the author—all demanded a wider circulation for the Essay than it could ever hope to attain in the original volume of Transactions where it is published. We have, therefore, endeavoured to condense all its important features within a reasonable compass, and hope it may excite a greater attention to the subject than has yet been paid to it.

